# Zn-DTSM, A Zinc Ionophore with Therapeutic Potential for Acrodermatitis Enteropathica?

**DOI:** 10.3390/nu11010206

**Published:** 2019-01-21

**Authors:** Lisa Bray, Irene Volitakis, Scott Ayton, Ashley I. Bush, Paul A. Adlard

**Affiliations:** The Florey Institute of Neuroscience and Mental Health, The University of Melbourne, 30 Royal Parade, Parkville, Victoria 3052, Australia; lisa.bray@florey.edu.au (L.B.); irene.volitakis@florey.edu.au (I.V.); scott.ayton@florey.edu.au (S.A.); ashley.bush@florey.edu.au (A.I.B.)

**Keywords:** acrodermatitis enteropathica, zinc, Zn-DTSM, therapeutic

## Abstract

Acrodermatitis enteropathica (AE) is a rare disease characterised by a failure in intestinal zinc absorption, which results in a host of symptoms that can ultimately lead to death if left untreated. Current clinical treatment involves life-long high-dose zinc supplements, which can introduce complications for overall nutrient balance in the body. Previous studies have therefore explored the pharmacological treatment of AE utilising metal ionophore/transport compounds in an animal model of the disease (conditional knockout (KO) of the zinc transporter, *Zip4*), with the perspective of finding an alternative to zinc supplementation. In this study we have assessed the utility of a different class of zinc ionophore compound (zinc diethyl bis(N4-methylthiosemicarbazone), Zn-DTSM; Collaborative Medicinal Development, Sausalito, CA, USA) to the one we have previously described (clioquinol), to determine whether it is effective at preventing the stereotypical weight loss present in the animal model of disease. We first utilised an in vitro assay to assess the ionophore capacity of the compound, and then assessed the effect of the compound in three in vivo animal studies (in 1.5-month-old mice at 30 mg/kg/day, and in 5-month old mice at 3 mg/kg/day and 30 mg/kg/day). Our data demonstrate that Zn-DTSM has a pronounced effect on preventing weight loss when administered daily at 30 mg/kg/day; this was apparent in the absence of any added exogenous zinc. This compound had little overall effect on zinc content in various tissues that were assessed, although further characterisation is required to more fully explore the cellular changes underlying the physiological benefit of this compound. These data suggest that Zn-DTSM, or similar compounds, should be further explored as potential therapeutic options for the long-term treatment of AE.

## 1. Introduction

The identification of acrodermatitis enteropathica (AE) as a distinct disease entity was first made by Danbolt and Closs in 1942, who postulated that AE was a “primary affection of the intestinal tract” (reviewed in [1]). This rare disease typically manifests in the early postnatal period and is characterised by skin lesions, gastrointestinal distress (e.g., diarrhoea), growth retardation, and hair loss—with symptoms becoming exaggerated with time and ultimately fatal if left untreated. Despite some successes with the use of diiodoquinoline in the treatment of AE [2], further mechanistic clarity did not come until decades later when Moynahan [3] demonstrated that zinc supplementation was sufficient to reverse symptoms of the disease. With the subsequent demonstration that AE patients had a failure in the absorption of zinc in the intestine [4,5] it was clear that AE was a primary zinc deficiency. In support of this, later genetic studies in several families would reveal that the Zrt- and Irt-like protein 4 (*ZIP4*) gene (*SLC39A4*; mapped to human chromosomal region 8q24.3), which has been shown to play a role in zinc homeostasis and to be regulated by zinc levels in vivo [6,7], was likely to be critical in the pathogenesis of disease [8]. Indeed, the introduction of AE-associated missense mutations into the *ZIP4* gene in mice resulted in decreased cellular uptake of zinc [9]. These cumulative discoveries around the pathogenesis of AE, which have been extensively reviewed (e.g., [10]), have led to the long-term treatment for AE being life-long high-dose zinc supplementation [11]. Whilst this approach has been effective, the use of zinc supplementation (in healthy individuals, as well as in those with zinc deficiency disorders such as AE) could lead to a number of adverse effects, such as deficiencies in other key nutrients such as copper [12,13,14,15]. Indeed, AE patients can be faced with a “juggling act” to maintain nutritional balance and to avoid subsequent clinical manifestations.

In order to further understand disease mechanisms and explore potential therapeutic options, a number of mouse models of AE have been proposed. Amongst these is a tamoxifen-inducible *Zip4*-enterocyte knockout model recently developed by Geiser and colleagues [16]. The primary phenotype of these animals involves a dramatic loss of weight following the induction of the intestinal *Zip4* knockout (KO), resulting from a switch from an anabolic to catabolic state in these mice, with the animals experiencing significant loss of muscle and bone mass prior to death within a matter of weeks. This model has been utilised to demonstrate that the *Zip4* gene controls animal growth and viability, impacts upon metal ion homeostasis, and results in a host of other biochemical, pathway, and anatomical changes that can drive the AE phenotype. Furthermore, it has also been used to assess therapeutic options. In this regard, we examined the utility of the 8-hydroxy quinoline compound, clioquinol (CQ), and found that when administered in concert with zinc it was highly effective at improving the phenotype in this model, in contrast to zinc and CQ alone which were ineffective [17]. This study represented a proof of principle investigation into the use of compounds that have been variably referred to as “chelators”, “chaperones”, “ionophores”, and “metal–protein attenuating compounds”. Whilst the nomenclature has varied, this broad class of compounds have been demonstrated to facilitate the redistribution of metals within the brain and to improve the phenotype in animal models of ageing, Alzheimer’s disease (AD), and Huntington’s disease (HD) [18,19,20,21,22,23,24,25,26,27,28,29,30,31]. The historical long-term use of CQ in human populations, however, has been associated with a number of potential significant side effects that has limited the ongoing use of CQ. As such, safer and potentially more potent metal targeting compounds have been developed. In this study we sought to assess the metal ionophore capacity, and potential efficacy of one such compound that belongs to a different chemical family to CQ, zinc diethyl bis(N4-methylthiosemicarbazone) (Zn-DTSM; Collaborative Medicinal Development), in the AE model. We investigated the use of a high (30 mg/kg/day) dose of the compound in young (1.5-month) and older (5-month) AE mice, in addition to a lower (3 mg/kg/day) dose of compound in the older AE mice. We also assessed metal levels in a number of different organs, including the brain, to determine whether there were any gross changes in metal levels in key tissues. Our data demonstrate that this compound was effective in improving the phenotype in the mice and could be considered for more targeted zinc delivery (with the potential benefit of reducing excess zinc exposure that may occur through bulk dietary zinc supplementation) to improve the long-term outcomes in AE patients.

## 2. Materials and Methods

### 2.1. Compound

**Figure 1:**
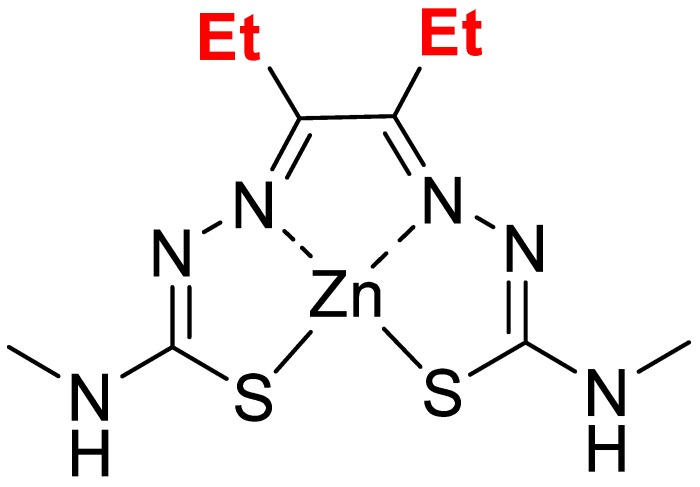
Chemical structure for zinc diethyl bis(N4-methylthiosemicarbazone) (Zn-DTSM).

The compound, Zn-DTSM, was a kind gift from Collaborative Medicinal Development. The chemical structure of this compound (Mw 351.8) is shown in Figure 1.

### 2.2. Ionophore Assay and Metal Analyses

The ionophore assay, which we have previously published on [20], utilised SH-SY5Y cells cultured in Dulbecco’s Modified Eagle Medium (DMEM) with 10% serum in this study. The compound/treatments were added for 24 h and the cells then washed in phosphate buffered saline (PBS), harvested and measured for metal content using inductively coupled plasma mass spectrometry (ICPMS). Both metal and compound were used at 10 µM, and repeat assays performed in triplicate.

We have previously published the ICPMS methods. Briefly, tissue samples were lyophilised and then digested with nitric acid (65% Suprapur, Merck, St. Louis, MO, USA) overnight, followed by heating at 90 °C for 20 min using a heat block. Samples were then removed from the heat block and an equivalent volume of hydrogen peroxide (30% Aristar, BDH, Radnor, PA, USA) added to each sample. Once samples had finished digesting, they were heated for a further 15 min at 70 °C. Samples were then diluted with 1% nitric acid diluent. Measurements were made using an Agilent 7700 series ICPMS instrument under routine multi-element operating conditions using a helium reaction gas cell. The instrument was calibrated using 0, 5, 10, 50, 100 and 500 ppb of certified multi-element ICPMS standard calibration solutions (ICP-MS-CAL2-1, ICP-MS-CAL-3, and ICP-MS-CAL-4, Accustandard, New Haven, CT, USA) for a range of elements, and we also utilised a certified internal standard solution containing 200 ppb of Yttrium (Y89) as a control (ICP-MS-IS-MIX1-1, Accustandard).

### 2.3. Ethics Statement

All animal experiments were approved by the Howard Florey Animal Ethics Committee (AEC#14-055) and were conducted in accordance with the Australian Code of Practice for the Care and Use of Animals for Scientific Purposes as described by the National Health and Medical Research Council of Australia. All animals had free access to food and water and were group-housed in individually ventilated cages in The Florey Institute Animal Facility under controlled temperature (22 ± 2 °C) and lighting (14:10 h light/dark cycle) conditions.

### 2.4. Animals and Experimental Details

We utilised the conditional *Zip4* (*Slc39a4*) KO mouse model of AE, which we have previously described [17] and which were a generous gift from Glen Andrews. These animals have a profound weight loss phenotype that manifests in the days following the induction of the *Zip4* KO and which will ultimately result in the death of the animal if left untreated. In all the experiments described herein animals (equally split between male and female) were weighed daily (a primary experimental endpoint) and were culled prior to reaching a terminal endpoint. For the induction of the KO, a tamoxifen stock solution was prepared by the addition of 100 µL of ethanol to 10 mg of tamoxifen (free base: MP Biomedicals, LLC, Santa Ana, CA, USA) and heating to 37 °C briefly to dissolve the tamoxifen. This solution was then diluted to 1 ml with autoclaved canola oil and heated briefly to 37 °C. The stock solution (10 mg tamoxifen/mL) was stored at 4 °C for up to two weeks or at −80 °C for longer-term storage. Before injection, the tamoxifen stock solution was heated to 37 °C. Mice were injected with 100 µL (intraperitoneal, 1 mg tamoxifen) of the tamoxifen stock solution daily for 3 consecutive days. The weight taken on the first day prior to the initial tamoxifen injection was used as the reference weight for each animal, and the subsequent weight change in response to the KO and/or the various treatments was calculated as a % weight change from this reference weight for each animal. The average % weight change was then calculated across the different groups and presented in the Figures. Following three days of rest, animals were then randomly assigned to specific experimental groups. Within this body of work there were three separate studies performed—Study#1, Study#2, and Study#3. Across these we did not examine the impact of Zn-DTSM in ”normal” animals (i.e. naïve mice with normal *Zip4* expression), as the compound is designed to restore a zinc deficit, and thus, in the absence of a zinc deficiency it is unlikely that we would see any compound effect. However, the effect of the compound in naïve animals would need to be assessed prior to any clinical advancement.

Study#1—following the post-tamoxifen rest period, AE animals (aged ~1.5 months) were given access to either water alone (“Control”, *n* = 6), water supplemented with zinc sulphate (250 mg/L) (“Zinc”, *n* = 6), or were orally gavaged with either Zn-DTSM (30 mg/kg/day made up in standard suspension vehicle (SSV), comprising 0.9% NaCl, 0.5% Na-carboxymethylcellulose, 0.5% benzyl alcohol, 0.4% Tween-80) and given access to water alone (“Zn-DTSM”, *n* = 6), or gavaged with Zn-DTSM and given water similarly supplemented with 250 mg/L zinc sulphate (“Zn-DTSM+Zinc”, *n* = 6) (note—all “Control” and “Zinc” AE animals also received daily oral gavages with SSV alone to properly control for the drug treatment groups). Following a week of treatment, animals were deeply anaesthetised using sodium pentobarbitone (100 mg/kg; diluted 1:10 in 0.9% saline), perfused with 25–30 mL of cold PBS (pH 7.4; diluted in 0.1M PBS) and then tissue harvested for metal analysis. The brain was micro-dissected into hippocampus, cortex, cerebellum and “remainder” of the brain; the proximal small intestine was flushed with cold PBS, cut into small 1-cm pieces; and the liver was similarly collected. All tissues were then frozen at −80 °C for biochemical analysis.

Study#2—This study was similar to Study#1, except the animals were ~5 months of age at the time of cull, and there were “Control” and “Zn-DTSM” groups (*n* = 5 and 4, respectively). We also included a group of naïve animals that were not treated with tamoxifen or in any other way manipulated, and these animals also had access to normal food and water (“Normal”, *n* = 3).

Study#3—This study was similar to that outlined in Study#1, with the following exceptions. The animals were all aged ~5 months in this study and the dose of Zn-DTSM was 3 mg/kg/day. The groups included were “Normal”, “Control”, “Zinc”, “Zn-DTSM” and “Zn-DTSM+Zinc” (*n* = 3, 9, 9, 9, 10, respectively). In the case of the groups receiving zinc supplementation, this was the same as shown above, with zinc sulphate spiked into the drinking water at 250 mg/L.

### 2.5. Statistical Analysis

**Figure 2:**
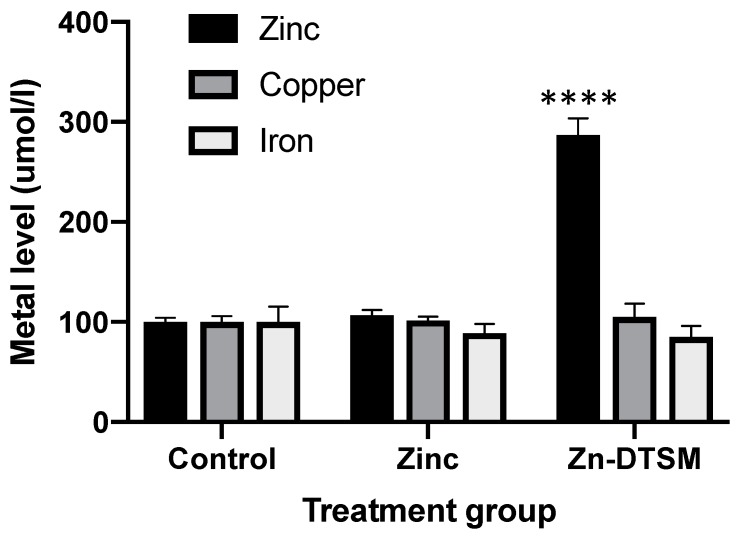
Ionophore data showing that cellular metal content in SH-SY5Y cells is not significantly altered by increasing exogenous zinc levels (10 µM), but the addition of 10 µM zinc diethyl bis(N4-methylthiosemicarbazone) (Zn-DTSM) significantly increases intracellular zinc. There is no significant effect of Zn-DTSM on either copper or iron levels. Data are mean ± SEM. ****p < 0.0001.

**Figure 3:**
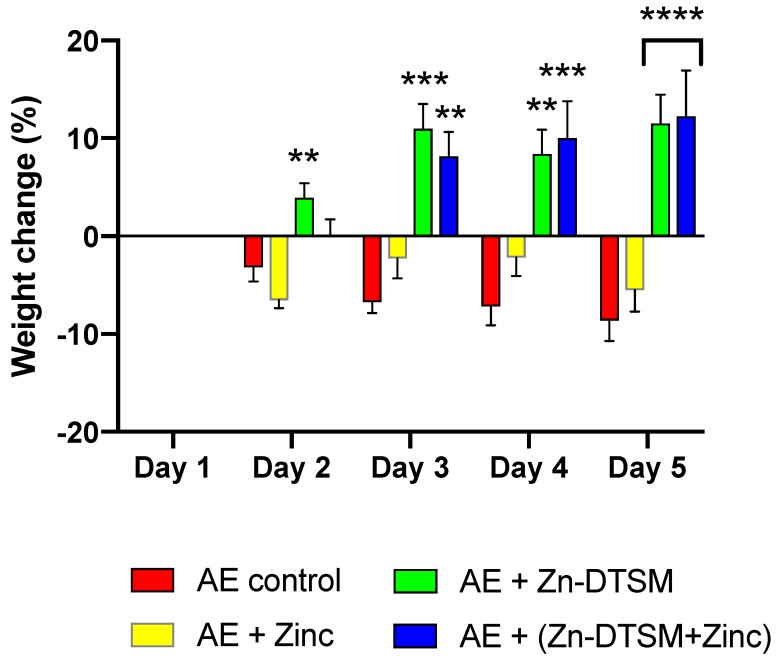
Weight change data (%) for young (1.5-month) acrodermatitis enteropathica (AE) animals treated with Zn-DTSM (30 mg/kg/day). There was an overall significant effect of treatment group (RM ANOVA, *p* < 0.0001) and also a significant interaction effect (*p* < 0.0001) across the study. The AE control animals show the expected weight loss across the five days of the experiment, which is marginally offset with dietary zinc supplementation. In contrast, the administration of either Zn-DTSM alone, or in combination with dietary zinc supplementation, results in a profound shift in the phenotype such that the animals gain significant amounts of weight over the time course. Data are mean ± SEM. **p < 0.01, ***p < 0.001, ****p < 0.0001 (all compared to AE+Zinc; on Day 5, both columns are significantly different to the AE+ Zinc group).

**Figure 4:**
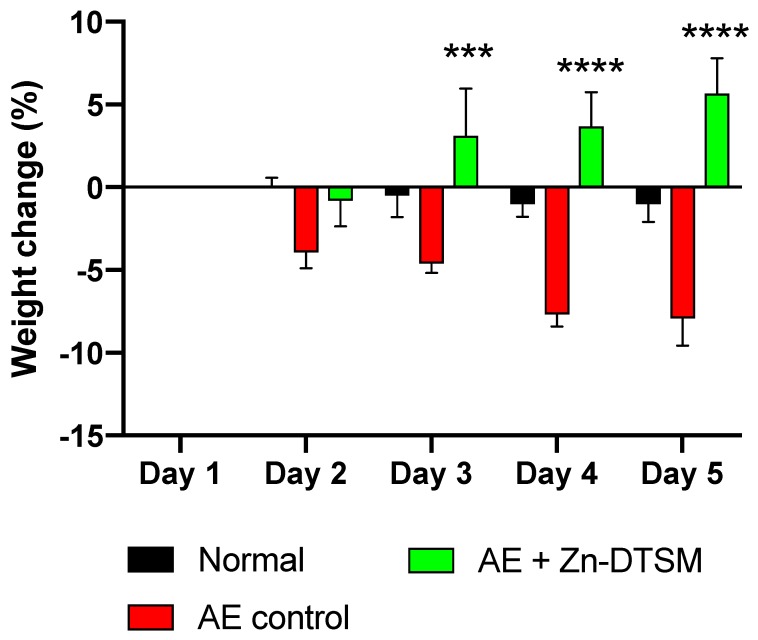
Weight change data (%) for older (5-month) AE animals treated with Zn-DTSM (30 mg/kg/day). These older AE control animals again lose weight as expected across the five days of the experiment, and this is significantly different to the naïve animals (“normal”) on days 2–3 (*p* < 0.05) and days 4–5 (*p* < 0.001). The administration of Zn-DTSM alone results in a profound shift in the phenotype such that the animals gain significant amounts of weight over the time course. Data are mean ± SEM. ***p < 0.001, ****p < 0.0001 (all compared to AE control).

**Figure 5:**
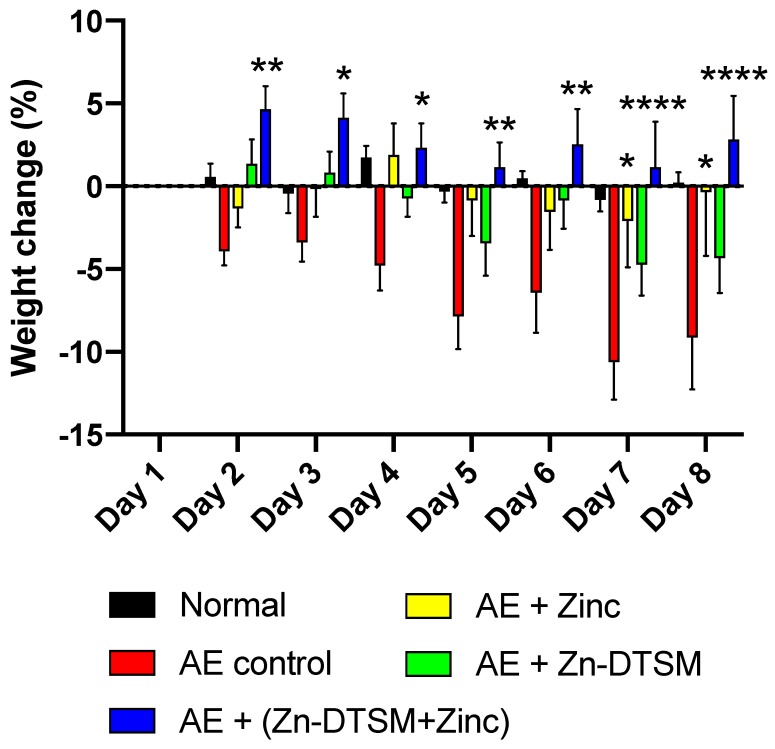
Weight change data (%) for older (5 month) acrodermatitis enteropathica (AE) animals treated with zinc diethyl bis(N4-methylthiosemicarbazone) (Zn-DTSM; 3 mg/kg/day). The AE control animals lose weight as expected across the time course (significantly different to the naïve “Normal” group on Days 7–8 (*p* < 0.05)), which is marginally offset by the provision of exogenous dietary zinc and also by the administration of Zn-DTSM (3 mg/kg/day) alone. The only intervention that is sufficient to significantly improve the weight loss phenotype consistently is the combination of the Zn-DTSM treatment and the zinc supplementation. Zinc treatment alone was significantly beneficial on Day 7 and Day 8 only. Data are mean ± SEM. *p < 0.05, **p < 0.01, ****p < 0.0001 (compared to the AE control group).

**Figure 6:**
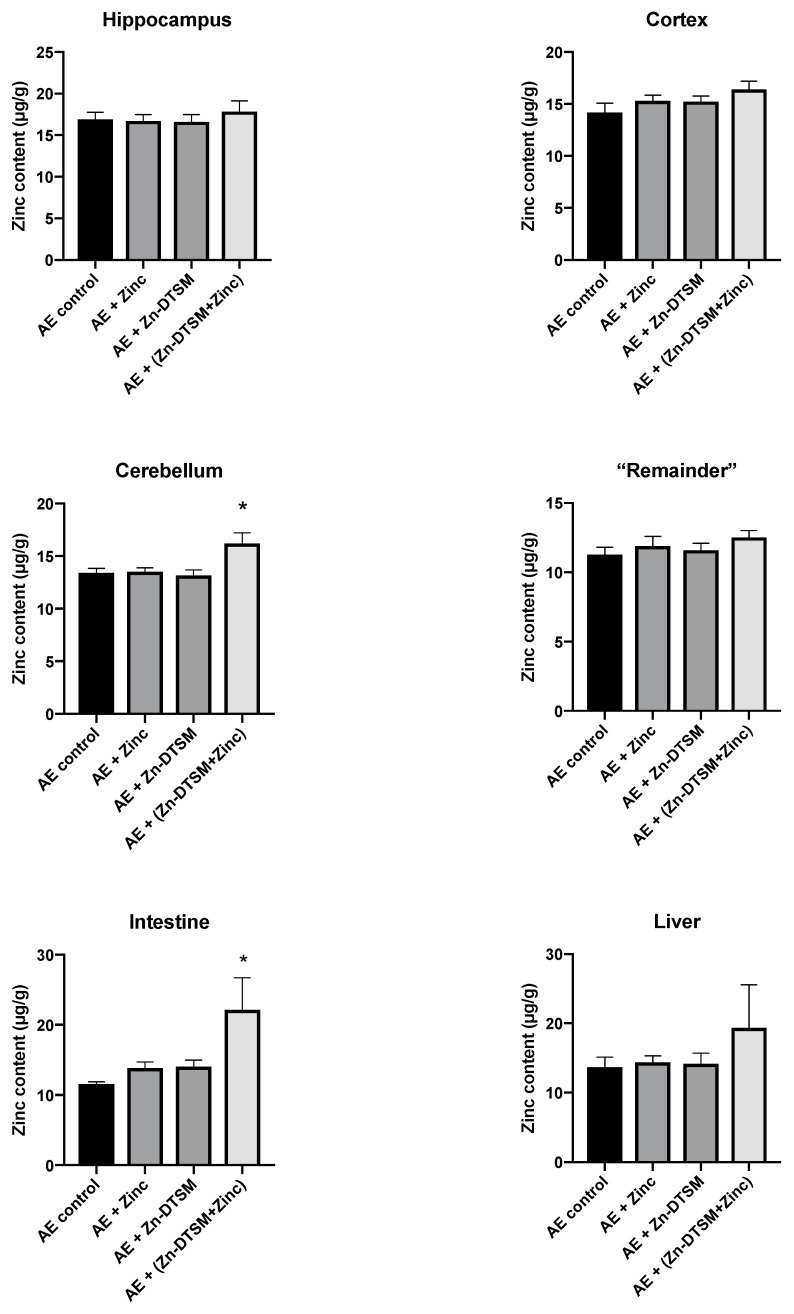
Zinc levels in various tissues harvested from 1.5-month-old acrodermatitis enteropathica (AE) mice treated with zinc diethyl bis(N4-methylthiosemicarbazone) (Zn-DTSM; 30 mg/kg/day). In the cerebellum there is a significant difference between the AE+(Drug+Zinc) group and all other groups (*p* < 0.05). Similarly, in the intestine, the AE+(Drug+Zinc) group is significantly different to the AE control group only (*p* < 0.05). Data are mean ± SEM. *p < 0.05.

**Figure 7:**
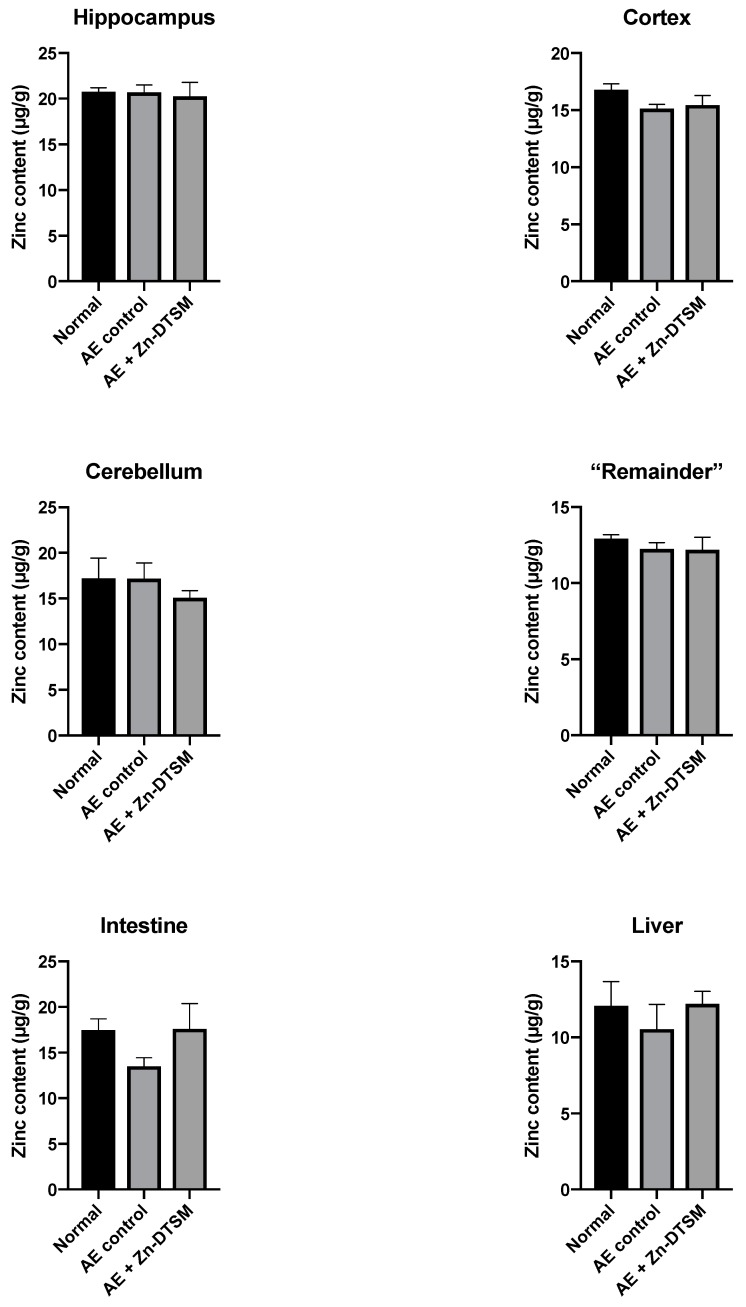
Zinc levels in various tissues harvested from 5-month-old acrodermatitis enteropathica (AE) mice treated with zinc diethyl bis(N4-methylthiosemicarbazone) (Zn-DTSM; 30 mg/kg/day). There are no significant effects of the drug on metal levels in any of the tissues examined. Data are mean ± SEM.

**Figure 8:**
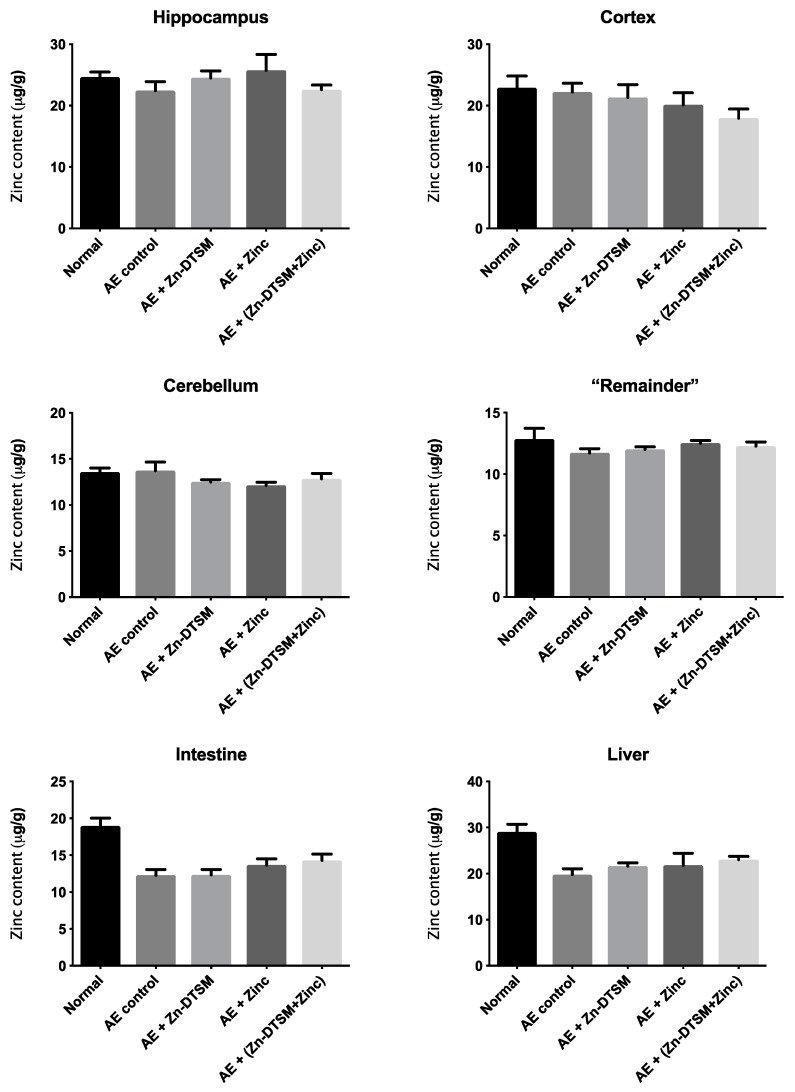
Zinc levels in various tissues harvested from 5-month-old acrodermatitis enteropathica (AE) mice treated with zinc diethyl bis(N4-methylthiosemicarbazone) (Zn-DTSM; 3 mg/kg/day). There are no significant effects of the drug, zinc supplementation or the combination of the drug+zinc on metal levels in any of the tissues examined. Data are mean ± SEM.

All statistical analyses were carried out using GraphPad Prism (version 8.0.0, San Diego, CA, USA). All statistical comparisons made are stated in the respective Figure Legends. For Figure 2, a two-way ANOVA with multiple post-hoc comparison tests was performed; for Figure 3, Figure 4 and Figure 5, a repeated measures (RM) two-way ANOVA with multiple post-hoc comparison tests was performed; for Figure 6, Figure 7 and Figure 8 a one-way ANOVA with multiple post-hoc comparison tests was performed.

## 3. Results

### 3.1. Zn-DTSM Is A Metal Ionophore

Consistent with what has been shown for other compounds, such as clioquinol and PBT2 [20], Zn-DTSM is a metal ionophore. There was an overall significant impact of the treatment group (*p* < 0.0001), with Zn-DTSM significantly increasing cellular zinc content (*p* < 0.0001; +287% compared to control at 100%; Figure 2). In contrast to the other compounds mentioned above, however, Zn-DTSM did not significantly alter the levels of the other metals measured (iron and copper). These in vitro zinc ionophore data are consistent with the apparent protective effect of the compound in the AE mouse model, as subsequently shown in Figure 3 and Figure 4, but it is not reflected in any substantive tissue level metal changes, as shown in Figure 6, Figure 7 and Figure 8. As noted in the discussion, however, this may be due to the acute nature of the experiment or the lack of clarity provided by the relatively “gross” tissue-level metal measurements.

### 3.2. Zn-DTSM (30 mg/kg/d) Prevents Weight Loss in Young (1.5-Month) and Older (5-Month) AE Mice

The “high” dose of Zn-DTSM was tested in mice in which the ablation of Zip4 was induced in young mice (~1.5 months of age). These data demonstrate that there was an overall significant effect of treatment group (RM ANOVA, *p* < 0.0001) and also a significant interaction effect (*p* < 0.0001) across the study (Figure 3). The main comparison for this study was to compare the effect of zinc treatment, which represents the clinical “standard of care” for AE patients, with the drug treatment (in the presence/absence of exogenous zinc). In this context, there were significant differences between the “Zinc” and “Zn-DTSM” group on Day 2 (*p* = 0.006), Day 3 (*p* = 0.0002), Day 4 (*p* = 0.006), and Day 5 (*p* < 0.0001). In the AE “Control” group there were also two deaths, whereas in the AE “Zinc” and AE “Zn-DTSM” group there were no deaths. Significantly, there was no statistical difference between the AE mice treated with either Zn-DTSM alone, or Zn-DTSM plus exogenous zinc. This demonstrates that Zn-DTSM at this dose does not require additional dietary zinc supplementation.

The weight loss that occurs in these mice is reported to result from a zinc-deficiency related switch in anabolic to catabolic metabolism that results in a loss of muscle and bone mass. The weight gain observed following compound treatment in the current study, therefore, is most likely just a prevention of this process (which occurs by the delivery of zinc). We have, however, not assessed any specific endpoints that would address the mechanisms underlying this weight gain. Further studies would be required to address this, and to definitively show whether or not this compound had any effect on weight gain independent of *Zip4*, and also whether there was any chance that it may cause obesity under any condition.

We also aged mice to 5 months prior to the induction of the *Zip4* ablation to assess whether or not the loss of *Zip4* had a similar effect on weight in the older adult animals (Study#2). In this study we only assessed the effect of Zn-DTSM alone. As shown in Figure 4, these older control AE mice lost significant amounts of weight compared to “Normal” mice (RM ANOVA, interaction effect *p* = 0.0003; treatment effect *p* < 0.0001; “Normal” vs. “Control” significantly different on days 4 and 5, *p* = 0.005 and *p* = 0.003 respectively), and similarly to Study#1, Zn-DTSM was effective at preventing this weight loss on Days 3, 4, and 5 (*p* = 0.0004, *p* < 0.0001 and *p* < 0.0001, respectively). In this study there were no dietary zinc supplements provided, and the compound alone was again effective at preventing the *Zip4*-induced weight loss that characterises these AE mice. Whilst the “Normal” mice did not gain weight over this acute time course, in contrast to the “AE+(Zn-DTSM)” mice that gained ~5%, there was not a significant difference in gross weight between these groups at the end of the study (indeed, the “Normal” mice were still ~9% heavier). Longer studies would be required to determine whether or not the AE mice treated with compound continued to gain weight or stabilised around wildtype control levels. Similarly, to definitively establish whether or not the compound itself could have a specific effect on weight, independent of the effects of the ablation of *Zip4*, additional experiments would be required.

### 3.3. Low Dose Zn-DTSM (3 mg/kg/day) Requires Exogenous Zinc to Prevent Weight Loss in Older (5-Month) AE Mice

Given the data from the first two studies, we assessed the effect of Zn-DTSM in a final set of experiments (Study#3; Figure 5). In these older mice, there was a significant (RM ANOVA) effect of both day (*p* = 0.039) and treatment group (*p* < 0.0001) across the study. The control-treated mice all lost weight across the trial, and this was only altered by zinc treatment on days 7 and 8 (*p* = 0.01 and *p* = 0.01 respectively). The Zn-DTSM treatment alone appears to have reduced the severity of the weight loss, but this did not reach statistical significance. In contrast, the animals treated with the combination of Zn-DTSM and exogenous zinc all gained weight, and were significantly different to the controls on Days 2–8 (*p* = 0.007, *p* = 0.02, *p* = 0.039, *p* = 0.005, *p* = 0.005, *p* < 0.0001 and *p* < 0.0001 respectively), but were no different to zinc-alone treated mice.

### 3.4. Acute Zn-DTSM Treatment Has Modest Effects on Tissue Zinc Levels

As shown in Figure 6, Figure 7 and Figure 8, there was little effect of any of the treatments on the zinc levels in the various tissues examined in the AE mice. The only significant differences in zinc were observed in the young animals treated with the higher dose (30 mg/kg/day) of Zn-DTSM in addition to exogenous zinc. Specifically, this was present in the cerebellum (ANOVA, *p* = 0.01), with the AE+(Zn-DTSM+Zinc) group significantly (*p* < 0.05) different to the AE control group; the AE+(Zn-DTSM+Zinc) group was also different to the AE+Zinc group (*p* < 0.05) and the AE+(Zn-DTSM+Zinc) group was different to the AE+Zn-DTSM group (*p* < 0.05). Also in the young group, there was a significant effect (ANOVA, *p* = 0.02) in the intestine, with the AE+(Zn-DTSM+Zinc) group significantly different to the AE control group (*p* < 0.05).

## 4. Discussion

In this study we have demonstrated that the novel metal ionophore, Zn-DTSM, is effective at preventing weight loss in the animal model of AE. This effect was observed when the *Zip4* gene was ablated in both young (1.5-month) and older (5-month) mice. Significantly, and unlike CQ that we have published on previously [17], this effect was apparent even in the absence of additional exogenous zinc (in the 30mg/kg/day group). At lower doses (3 mg/kg/day) a significant beneficial effect of Zn-DTSM was only evident when exogenous zinc was supplied in the drinking water. These data open up new avenues for investigation into pharmacological approaches for the treatment of AE which may decrease, or even avoid, the requirement for daily high dose zinc supplementation.

The Zn-DTSM compound utilised in this study belongs to the bis thiosemicarbazone family of compounds. We have previously utilised compounds from this chemical class in other disease models, including Cu(GTSM) that was utilised in the APP/PS1 mouse model of AD [24]. In that study, the copper ionophore compound had profound effects on modulating the phenotype of those animals (including an inhibition of GSK3ß, a decrease in Aβ multimers and the phosphorylation of tau; and an improvement in cognitive performance). Together with other studies utilising metal ionophore compounds, such as CQ [32] and PBT2 [18,20] in AD models; PBT2 [31] and Cu(GTSM) [29] in a tauopathy model; and PBT2 in models of HD [23], ageing [22] and traumatic brain injury [30]—these reports demonstrate the potent nature of compounds that modulate metal ion homeostasis in various models of human disease.

In the case of AE, we have previously tested the efficacy of CQ [17] in the *Zip4* KO mouse model and shown that it is ineffective in preventing the phenotype present in this model when given in isolation. However, in the presence of exogenous zinc, then the compound was highly effective in preventing the rapid weight loss that leads to eventual death in this model. In that study we also interrogated potential mechanisms of action of CQ and demonstrated significant effects on intestinal cells. Given the marginal zinc ionophore capacity of CQ [20] it is perhaps not surprising that it did not work in this model in the absence of exogenous zinc. In contrast, the Zn-DTSM compound used in the current study is a primary zinc ionophore, resulting in a near three-fold increase in cellular zinc levels (Figure 2). This is similar to PBT2, which is a far more potent zinc ionophore than CQ and has demonstrated an ~2 fold increase in zinc when used at the same concentration as Zn-DTSM [20].

Consistent with its capacity for zinc delivery, Zn-DTSM (30 mg/kg/day) was shown to be far more effective in preventing the primary weight loss phenotype in the AE model than CQ. Importantly, the effect of Zn-DTSM was achieved in the absence of any exogenous zinc and it was also beneficial in both young and older AE animals, suggesting that it may be of potential benefit to both juvenile and adult-onset AE. However, our studies were acute in nature (to avoid the lethal phenotype in this model), and so more chronic studies are required to investigate the longer-term benefit of Zn-DTSM and to also understand the mechanism of action of this compound in this disease. Such studies will also be valuable to tease apart the dose–response for the compound, as we also assessed a lower dose of compound (3 mg/kg/day) in older mice and found that it was only effective at preventing significant weight loss when given in the presence of exogenous zinc, and even then it was no more beneficial than zinc alone. Thus, understanding the limiting dose of compound, from the perspective of the requirement for exogenous zinc supplementation, will be important from a clinical and drug development perspective. This will also allow us to further interrogate the effect of these compounds on the metalloproteomic profile of the mice.

Specifically, ZN-DTSM had no apparent effect on either copper or iron levels in the cell (in the ionophore assay), whereas other 8-hydroxy quinoline compounds (such as PBT2) can have a significant ionophore capacity for other metals such as copper [20]. This apparent specificity of Zn-DTSM (a bis thiosemicarbazone) for zinc may have some clinical benefit by limiting the off-target effects that may arise from treatment with specific compounds (which may alter the cellular content of various metals other than zinc) or from the typical high-dose zinc supplementation that is used clinically (which can also directly impact other metal species such as copper). Indeed, the ICPMS studies conducted here revealed surprisingly little effect of Zn-DTSM treatment on the zinc content of various tissues that were harvested from the AE animals for metal analysis, although clearly longer-term studies and perhaps also a finer interrogation of metal levels in more specific cellular compartments, are required to further elucidate this. In this regard, the analyses conducted here were also undertaken as a preliminary investigation into the effect of the induction of AE on brain metal levels. Perhaps not surprisingly, again given the acute nature of the experiments, we did not observe any change in metal levels in the various brain regions examined (hippocampus, cortex, cerebellum and the “rest”) following the ablation of *Zip4*. Longer-term studies will allow us to assess whether the AE-associated zinc insufficiency translates to CNS deficits that then impact/translate to impairments in higher order functions such as learning and memory, or contribute to other metal-associated psychiatric manifestations.

## 5. Conclusions

These studies demonstrate that Zn-DTSM is a potent zinc ionophore that may have utility in the treatment of zinc deficiency disorders, such as AE. This, or similar compounds, may eliminate the need for the daily high-dose zinc supplementation that is used clinically, and which may contribute to a long-term disruption in normal nutrient balance in the body that can potentiate other undesirable clinical symptoms. Additional studies are required, however, to address the long-term consequences of treatment with this compound in both naïve and disease animals. Furthermore, understanding the impact of chronic zinc deficiency (in the AE animals) on other physiological parameters, such as learning and memory, is critical given the known role of zinc in these higher order cognitive processes. Whether zinc ionophores may also have some role in such situations also remains an open question that should be investigated.
